# The Effect of Virus-Blocking *Wolbachia* on Male Competitiveness of the Dengue Vector Mosquito, *Aedes aegypti*

**DOI:** 10.1371/journal.pntd.0003294

**Published:** 2014-12-11

**Authors:** Michal Segoli, Ary A. Hoffmann, Jane Lloyd, Gavin J. Omodei, Scott A. Ritchie

**Affiliations:** 1 Mitrani Department of Desert Ecology, The Jacob Blaustein Institutes for Desert Research, Ben-Gurion University of the Negev, Midreshet Ben-Gurion, Israel; 2 School of Public Health and Tropical Medicine and Rehabilitative Sciences, James Cook University, Cairns, Queensland, Australia; 3 Bio21 Institute, Department of Genetics, The University of Melbourne, Parkville, Victoria, Australia; University of Perugia, Italy

## Abstract

**Background:**

The bacterial endosymbiont *Wolbachia* blocks the transmission of dengue virus by its vector mosquito *Aedes aegypti*, and is currently being evaluated for control of dengue outbreaks. *Wolbachia* induces cytoplasmic incompatibility (CI) that results in the developmental failure of offspring in the cross between *Wolbachia*-infected males and uninfected females. This increases the relative success of infected females in the population, thereby enhancing the spread of the beneficial bacterium. However, *Wolbachia* spread via CI will only be feasible if infected males are sufficiently competitive in obtaining a mate under field conditions. We tested the effect of *Wolbachia* on the competitiveness of *A. aegypti* males under semi-field conditions.

**Methodology/Principal Findings:**

In a series of experiments we exposed uninfected females to *Wolbachia*-infected and uninfected males simultaneously. We scored the competitiveness of infected males according to the proportion of females producing non-viable eggs due to incompatibility. We found that infected males were equally successful to uninfected males in securing a mate within experimental tents and semi-field cages. This was true for males infected by the benign *w*Mel *Wolbachia* strain, but also for males infected by the virulent *w*MelPop (popcorn) strain. By manipulating male size we found that larger males had a higher success than smaller underfed males in the semi-field cages, regardless of their infection status.

**Conclusions/Significance:**

The results indicate that *Wolbachia* infection does not reduce the competitiveness of *A. aegypti* males. Moreover, the body size effect suggests a potential advantage for lab-reared *Wolbachia*-males during a field release episode, due to their better nutrition and larger size. This may promote *Wolbachia* spread via CI in wild mosquito populations and underscores its potential use for disease control.

## Author Summary

Dengue is a tropical, potentially lethal disease transmitted by mosquitoes. A new control method involves the release of mosquitoes infected by the bacterium *Wolbachia* that blocks the transmission of the dengue virus to humans. However, possible negative effects of *Wolbachia* on mosquito reproductive success could substantially slow the spread of this bacterium in mosquito populations, reducing the feasibility of this method. We found that male mosquitoes infected by *Wolbachia* are equally successful in finding and mating with females within experimental tents and semi-field cages that mimic mosquito natural habitat. Moreover, larger, well-fed mosquitoes were more successful in semi-field cages, suggesting that *Wolbachia* mosquitoes that are reared in the lab, and are generally larger than wild mosquitoes, might have an advantage during the time they are being released in the field. Hence, in contrast to other control methods (*e.g.*, the use of sterile males or genetically modified mosquitoes), the use of *Wolbachia* does not seem to compromise male performance, making it a candidate for disease control.

## Introduction

Vector-borne diseases are infections transmitted by the bite of arthropod species, primarily mosquitoes. These diseases (*e.g*., malaria, dengue, Chagas disease and filariasis) are major contributors to human mortality and morbidity, especially in developing tropical countries. Traditional control measures including the use of vaccines to reduce pathogen development or the use of insecticides to suppress the vector population are often not sufficient. Hence, there is an urgent need for novel approaches [1]. Over the years researchers have been developing a range of alternative control strategies aimed at the suppression or replacement of the mosquito vector population via mass-releases of modified mosquitoes. Modifications include sterilization of males to reduce reproduction of wild females [2]; genetic modifications to introduce lethal genes [3] or genes that reduce disease transmission [4] into wild mosquito populations; and infection of the mosquitoes by a second agent such as the bacterium *Wolbachia*, to suppress pathogen transmission [5]. Despite their potential, the success of these methods is dependent on the ability of released mosquitoes to survive and reproduce in the field. For example, for the success of the sterilization technique it is crucial to ensure that sterilized males are sufficiently competitive and attractive to wild females [6]. Similarly, transgenic mosquitoes should be able to survive and mate in the field in order to introduce novel genes into the population [7]. Finally, mosquitoes infected by a bacterial agent should be successful enough to allow it to spread and establish in wild populations [8]. Therefore, each development should be accompanied by careful assessments of potential fitness effects.

Dengue is a viral tropical disease that affects hundreds of million of people throughout the world [9], [10]. Although dengue is normally not life threatening, its complications may be lethal. In addition, dengue inflicts enormous economical and social burdens [11], [12]. Dengue is transmitted primarily by the mosquito *Aedes aegypti* that is highly adapted to human habitats [13]. Due to a combination of ecological and anthropological conditions, the prevalence, distribution and impacts of dengue are currently increasing [14].

One of the most promising developments for dengue control focuses on the release of lab-reared mosquitoes that are infected by the bacterium *Wolbachia pipientis* to reduce the transmission of dengue virus. *Wolbachia* is a maternally transmitted intracellular bacterium that naturally occurs in many insect species, including mosquitoes. Although *Wolbachia* does not occur naturally in *A. aegypti*, *Wolbachia* strains derived from *Drosophila melanogaster* were artificially introduced by embryo microinjection into laboratory lines [15], where it was shown to suppress the development of the dengue virus [16], [17]. In addition, *Wolbachia* induces cytoplasmic incompatibility (CI) that results in the developmental failure of offspring in the cross between uninfected females and *Wolbachia*-infected males. This increases the relative success of infected females in the population, thereby enhancing the spread of the bacterium [18]. The combination of virus blockage and the ability of *Wolbachia* to invade mosquito populations make this intervention a prime candidate for dengue control [5], [19], [20]. The method relies on the release of both *Wolbachia* infected females (to ensure *Wolbachia* transmission) and males (to ensure *Wolbachia* spread via CI). However, *Wolbachia* spread via CI will only be feasible if infected males are sufficiently competitive in obtaining a mate under field conditions.

*Wolbachia* was found to induce several fitness effects in *A. aegypti* including reduced lifespan [8], [15], reduced fecundity [19], [21], reduced ability to feed [22], reduced egg viability [8], [23], increased locomotor activity and increased or decreased metabolism (depending on sex and age) [24]. The magnitude of these fitness effects may determine whether a particular *Wolbachia* strain will spread to fixation, or disappear from the population following a release [25]. Therefore, understanding these effects is important for making strategic decisions regarding the number and density of mosquitoes to be released. However, so far many of the fitness tests were conducted under laboratory conditions, and may not be relevant under realistic field conditions [26]. In particular, the effect of *Wolbachia* on *A. aegypti* male competitiveness under field or semi-field conditions has not yet been tested, despite its importance in generating CI that allows the infection to spread [18]. There are several reasons to assume *Wolbachia* might affect male competitiveness. First, *Wolbachia* may potentially affect male vigor and behavior directly, thereby altering the ability of males to secure females or altering their attractiveness to females. Second, males of infected colonies that were produced and reared under laboratory conditions may potentially suffer fitness costs due to reduced genetic diversity and the expression of inbreeding depression, although this may be avoided by backcrossing the colony with wild individuals [8]. On the other hand, during a field release, lab-reared *Wolbachia* males may have an advantage over wild males due to improved nutrition leading to an increase in male size. Larger *Aedes* males may transmit more sperm to females during mating [27] and be less prone to sperm depletion [28]. In addition, at least in *Anopholes* mosquitoes, larger males are more competitive [29] and more likely to acquire mates in the field.

Our goal was to compare the competitiveness of *A. aegypti* males infected by *Wolbachia* derived from *Drosophila melanogaster*, with that of uninfected males. We used mosquitoes infected by two *Wolbachia* strains: 1) the *w*Mel strain that has relatively mild fitness effects on its host, and an ability to block dengue transmission; and 2) the *w*MelPop (popcorn) strain that induces higher fitness costs but even stronger blockage of the dengue virus [19]. Mosquitoes infected by these strains have been previously used in release trials in Cairns, Northern Queensland, Australia, and its suburbs. The popcorn strain failed to establish and decreased in abundance following the releases, suggesting high fitness costs for infected mosquitoes under field conditions [30], which may or may not include reduced competitiveness for infected males. The *w*Mel strain established successfully in several locations in and around Cairns, suggesting lower fitness costs [20]. The ability of *w*Mel to invade large continuous populations of mosquitoes and to spread out of an initial release zones is yet to be determined and will depend on fitness costs [31].

In a series of experiments in tents and semi-field cages reflecting aspects of the natural habitat for *A. aegypti* mosquitoes, we exposed uninfected females simultaneously to *Wolbachia*-infected and uninfected males, and tested the competitiveness of infected males. In a complimentary experiment, we tested the combined effect of male size and infection status on the competitiveness of males.

## Methods

### Establishment of mosquito colonies and rearing

Colony establishment and rearing conditions were similar to other studies [19], [20]. The *Wolbachia* infected strains had been backcrossed to uninfected *A. aegypti* sourced from several locations around Cairns for multiple (>5) generations to ensure that genetic backgrounds were similar. The uninfected field mosquitoes were stored as eggs which were then hatched at different times to provide adult males for backcrossing. The *w*Mel infected line continued to be backcrossed just prior to the experiments, while the *w*MelPop infected line had not been backcrossed for a year in the period leading up to the experiments, but had been maintained at a large population size of several hundred individuals during this period. The performance of the *Wolbachia* infected stocks was compared to the F3 laboratory generation of Cairns *A. aegypti* sourced from the field. The uninfected line (F3) was established from wild *A. aegypti* collected from ovitraps set in 2013 in suburbs of Cairns where no releases of *Wolbachia* infected mosquitoes had occurred, and confirmed as being uninfected by mating females with *w*Mel infected males and by PCR.

Experiments were conducted from May 2013 till February 2014 at the James Cook University Mosquito Research Facility Semi-Field System in Cairns, Queensland, Australia [32]. Temperatures in the experimental semi-field cage ranged from ∼25°C in the colder months (May-September) to ∼30°C during the hotter months (October-February). To ensure virginity, males and females were separated during the pupal stage and were kept in cups (720 ml) containing 5–10 pupae. After emergence mosquitoes were given access to 50% honey solution, but honey was removed from cups with females prior to the beginning of the experiments (at least 24 hrs in exp. 1 and 48 hrs in experiments 2–3) as females engorged with honey were less likely to feed on blood. Males and females were 3–7 days old at the beginning of the experiments, except for few repetitions where males were older (see experiment 2, below). The age range of uninfected and infected males in each repetition was similar. The infection status of lines and mosquitoes was confirmed where possible through PCR assays [33].

### Experimental design—Competitiveness of infected vs. uninfected males

For each experimental repetition we placed 20 uninfected females, 15 *Wolbachia* infected and 15 uninfected males, in a tent (170×170 cm, with a maximum height of 190 cm; mosquito density  = ∼9 per m^2^) located within a large semi-field cage (Cage A, 17.5 m×8.7 mm, with a respective height of 2.8 m and 4.1 m at the wall and centre of the ceiling [32]). Each tent contained a potted plant and a 10 L bucket with 5 L standing water to mimic mosquito habitats in urban backyards and to induce swarming and mating behavior. As a control, we placed 20 uninfected females with 30 uninfected males in a tent (compatible cross), or 20 uninfected females with 30 infected males (incompatible cross), to evaluate baseline egg viability and incompatibility rates, and to evaluate our scoring method for viable vs. non-viable females. In addition, we ran several experimental replications (17 altogether) where mosquitoes were released directly into a large semi-field cage (Cage A; mosquito density ∼0.35 per m^2^), with 60 uninfected females, 45 uninfected and 45 infected males, or a smaller cage (Cage A subdivided; mosquito density ∼0.5 per m^2^) with 40 uninfected females, 30 uninfected and 30 infected males. While these densities are probably high in comparison to the estimated mean densities in the field (*e.g.*, 5–10 females per house-hold in Cairns [34]), field density can be locally high (*e.g*., up to 58 mosquitoes from a single BG sentinel trap/day during the wet season in Cairns [35]). The relative numbers of males and females were designed to induce male-competition while keeping the sex-ratio realistic. At 22–26 hr after the beginning of the experiment, a human subject entered each of the tents/cages for feeding mosquitoes (James Cook University Human Ethics Approval H4907). Blood-fed females were captured using a mechanical aspirator and placed into individual transparent plastic oviposition cups (11 cm height, 4.5 cm diameter). Each cup contained a strip of sandpaper along the bottom (oviposition substrate) and was filled with tap water to a depth of 1–3 cm. Seven days later, females were removed and a sample dissected (see ‘Mating success of experimental females’ below); sandpaper was clipped to the top of the oviposition cup to prevent contact with water, and eggs were incubated in this moist environment for additional seven days. Egg strips were then submerged to induce hatching. Eggs were counted 24 h later and scored for viability under a dissecting microscope. Hatched eggs were easily recognizable by the missing operculum. Each unhatched egg was probed with an insect-pin to see whether it was empty or contained a larva. Eggs were scored as viable if they had hatched or contained a larva.

Due to CI induced by *Wolbachia*, uninfected female *A. aegypti* fail to produce viable offspring in the cross with infected males [15], [20]. This enabled us to estimate the competitiveness of infected males according to the proportion of non-viable females in a tent or a cage. Female *Aedes* are considered monogamous, but some degree of multiple mating may occur [36], [37]. Therefore, we considered a female as viable if it produced ≥50% viable eggs (indicating mating and use of sperm of an uninfected male), and as non-viable if it produced <50% viable eggs (indicating sperm of an infected male). We ran three experiments comparing the competitiveness of infected vs. uninfected males in tents and in semi-field cages (experiments 1–3). In addition we compared the survival of infected vs. uninfected males in small insect cages (experiment 4).

### Experiment 1—Competitiveness of wMel infected vs. uninfected males

We exposed uninfected females simultaneously to uninfected males, and to males infected by the *Wolbachia* strain *w*Mel (n = 6 repetitions of compatible controls, 5 incompatible controls, 18 repetitions in experimental tents, 1 run in the large cage and 3 in the subdivided cage).

### Experiment 2—Competitiveness of wMelPop infected vs. uninfected males

Uninfected females were exposed to uninfected males, and to males infected with *w*MelPop (n = 5 compatible controls, 5 incompatible controls, 12 experimental tents and 3 experimental cages subdivided). In addition, because some fitness effects imposed by the popcorn strain are mostly expressed later in life [8], [24], [38], we also ran three repetitions in the tents using infected and uninfected older (10–14 days) males. These males were not exposed to females to prevent sperm depletion.

### Experiment 3—Competitiveness of wMel infected vs. uninfected males of different sizes

To produce smaller males, we reared uninfected larvae and larvae infected by *w*Mel on ¼ the amount of food. Adult males reared on this diet took longer to develop. We compared body size, estimated as wing length [39] of these smaller males, to larger males and to wild males (trapped in the field during Nov-Dec 2012; [30]). While the effect of smaller size due to low nutrition may be essentially different than that of smaller size due to genetic tendency, we chose this manipulation as it better represents the difference between *Wolbachia* infected mosquitoes and wild mosquitoes during a release (*i.e.,* different nutrition, but similar genetic background).Female size was not manipulated (mean ± SD of wing length  = 2.87 mm ±0.18, based on a sample of 100 females) and was within the natural range (*e.g*., 2.65–2.96 [30]). To examine the combined effect of infection status and body size on male competitiveness, we placed 20 uninfected females, 15 infected and 15 uninfected males of a different size in each experimental tent (n = 6 repetitions with larger uninfected and 6 with larger *w*Mel infected). We used larger tents (300×300 cm, with a maximum height of 200 cm; mosquito density ∼3 per m^2^) with a finer mesh for these experiments, to prevent small males escaping. In the semi-field cage (cage A subdivided) we placed 40 females with 30 uninfected and 30 infected males of different sizes (n =  repetitions with larger uninfected and 5 with larger *w*Mel infected). We scored competitiveness according to the number of viable vs. non-viable females for each size/infection-status combination. We predicted that more females would produce viable (or non-viable) eggs when the larger males were uninfected (or infected).

### Female recapture and oviposition success

We were not always successful in feeding all the females in a tent/cage, and not all females in the experiments produced eggs. We suspect that this is representative of natural field conditions, where unfed females are found in traps after releases [30]. We excluded 15 females that laid fewer than 10 eggs, and scored a mean of 12.11 (SD 4.12, range 3–19) females in the experimental tents and 18.0 (SD 6.96, range 5–27) in the semi-field cages. The number of females producing eggs increased throughout the experimental period, probably due to conditions becoming warmer. The mean ± SD number of eggs laid per female was 53.38±22.89 in experiment 1, 71.46±.18 in experiment 2, and 65.40±28.69 in experiment 3.

### Mating success of experimental females

We dissected a sample of 1098 experimental females (∼85% of total) of both tents and cages to look for sperm. Dissections were conducted with a stereomicroscope and each spermatheca (each female has three) was observed for motile sperm under a phase contrast microscope (100x) to confirm that the production of non-viable eggs reflected incompatibility rather than female virginity. Only in three cases were sperm absent in any of the spermathecae of a female, suggesting extremely high mating (99.7% of females). Hence, we assumed all females were inseminated.

### Experiment 4—Male survival

A basic assumption in our experiments was that male survival is similar among males of different sizes or with a different infection status. Hence, potential differences in the proportion of viable vs. non-viable females were attributed to mating competitiveness rather than survival. To confirm this assumption, we placed 10 males of a comparable size and infection status, with or without 10 uninfected females, in a small insect cage (Bug Dorm, 30×30×30 cm), with access to water. We repeated each combination four times resulting in 4 repetitions x 2 male sizes x 2 infection status x 2 female presence  = 32 cages. Two cages failed (mosquitoes escaped). We recorded the number of dead males after 1 day and after 1 week.

### Statistical analyses

We used STATISTICA 7.0 (StatSoft inc.) to conduct the analyses. Because our research question focused on male competitiveness in securing a mate, we used females rather than their progeny as the data points. Hence, to assess the competitiveness of infected vs. uninfected males (Exp. 1–3), we compared the frequency of females that were scored as viable vs. non-viable (with the null hypothesis of 1∶1) using chi-square tests, considering each tent/cage as a repetition. We ran the analysis separately for control and experimental repetitions, and separately for females from experimental tents and from the cages. Due to the small number of females that laid eggs in some of the repetitions, we also ran these tests while excluding repetitions with fewer than 10 females. However, this did not change the statistical outcome; therefore we only present the full analysis that included all repetitions. In addition, we computed the Fried Competitiveness Index [40]:

C =  (w/s) × [(Hw − Hc)/(Hc − Hs)], where w and s  =  the number of competing uninfected and infected males, respectively, Hw  =  the percentage viable eggs in the compatible controls, Hc  =  the percentage viable eggs in the competitiveness trial and Hs  =  the percentage viable eggs in the incompatible controls. Index values were similar regardless of whether we used % viable females or % viable eggs and only the former are presented. We also used Fisher's exact test to compare the frequency of viable females among certain treatments (see results). We ran two-way ANOVAs to examine the combined effect of rearing conditions (high diet, low diet and wild) and infection status on male body size (estimated as wing length). For the survival experiment (Exp. 4), we ran a three-way ANOVA to test the combined effect of size-class, infection-status, and female presence on the number of dead males in a cage after a day and after a week.

### Ethics statement

Human Ethics Approval H4907 was provided by Human Research Ethics Committee, James Cook University (Human Ethics Advisor: Julie Parison; Head of Committee: Anne Swinbourne). All adult subjects provided informed oral consent (no children were involved). Names of subjects providing oral consents were recorded in writing. Written consents were not taken because this was not required by the ethic's committee.

## Results

### Competitiveness of wMel infected vs. uninfected males—Exp. 1

**Figure 1:**
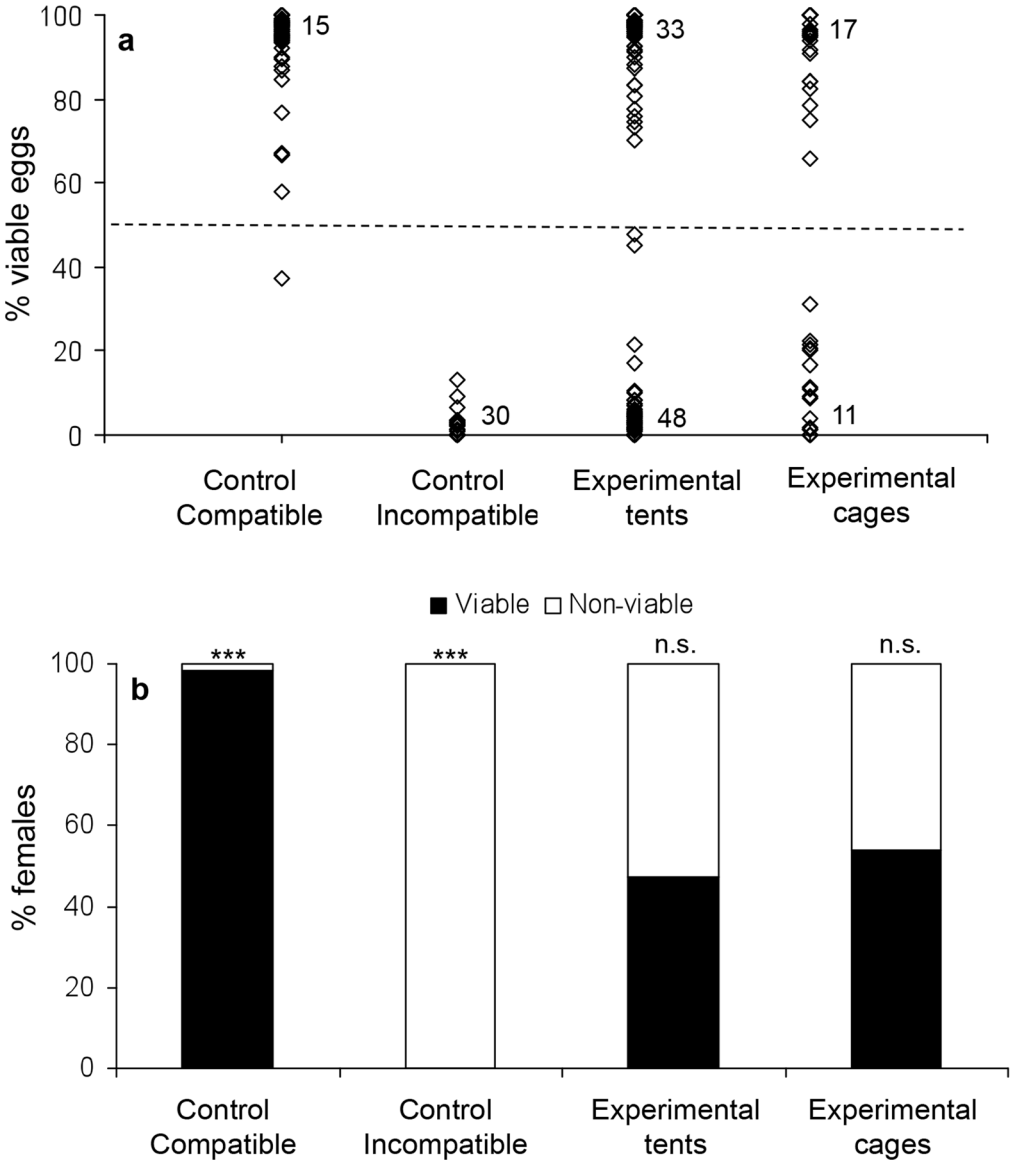
Experiment 1: Competitiveness of *w*Mel infected vs. uninfected males. Data pooled for all females of a certain treatment a) Percentage of viable eggs for females of compatible controls (n = 60 females), incompatible controls (n = 48 females), experimental tents (n = 161 females) and semi-field cages (n = 57 females). Numbers on bottom and top of figure represent the number of overlapping data points with extreme values (0% and 100% respectively) The dashed line represent the threshold for scoring females as viable (≥50%) or non-viable (<50%). b) Percentage of viable and non-viable females in each of the above treatments. Asterisks represent significance level for deviation from 1∶1 using observed vs. expected chi square test with each tent/cage as a repetition (see text).

The majority of females from the compatible control (n = 50 out of 60) produced over 90% viable eggs (range 37.5%–100%), and with the exception of a single female, all produced over 50% viable eggs (Fig. 1, χ^2^ = 28.14, df = 5, p<0.001, n = 6 repetitions in tents, 60 females in total). The occurrence of non-viable eggs in the compatible control was probably due to desiccation or other developmental failures unrelated to the expression of cytoplasmic incompatibility. The majority of females from the incompatible control (n =  out of 48) produced less than 10% viable eggs (range 0%–13.3%), and none produced over 50% (Fig. 1, χ^2^ = 24.0 df = , p<0.001, n =  tents, 48 females). The proportion of viable eggs in the incompatible control was higher than previously reported for incompatible crosses [8], [15]. This difference is likely to reflect methodologies, because unlike in previous studies we only hatched eggs for 24 hours, and inferred viability also based on the presence of larvae within the eggs. Some of these larvae might have already been dead or carrying developmental defects due to cytoplasmic incompatibility. This would have led to an overestimation of the proportion of viable eggs in incompatible crosses, but should not affect interpretations because there was no overlap in the proportion of viable eggs between the compatible and non-compatible controls.

The majority of females in the experimental tents and cages (188 out of 218) produced >90% or <10% viable eggs, suggesting low levels of multiple mating. The results of the chi square test considering each trial as a repetition indicated no deviation from 50% in the percentage of viable vs. non-viable females in the experimental tents (Fig. 1, χ^2^ = 13.81, df = , p = .68, n = 18 tents, 161 females), or in the experimental cages (Fig. 1, χ^2^ = 3.16, df = 3, p = 0.37, n = 4 cages, 57 females) suggesting similar competitiveness for *w*Mel infected and uninfected males under the experimental conditions used. The competitiveness index of infected vs. uninfected males was 1.06 for males competing in the tents and 0.70 in the cages.

### Competitiveness of wMelPop infected vs. uninfected males—Exp. 2

**Figure 2:**
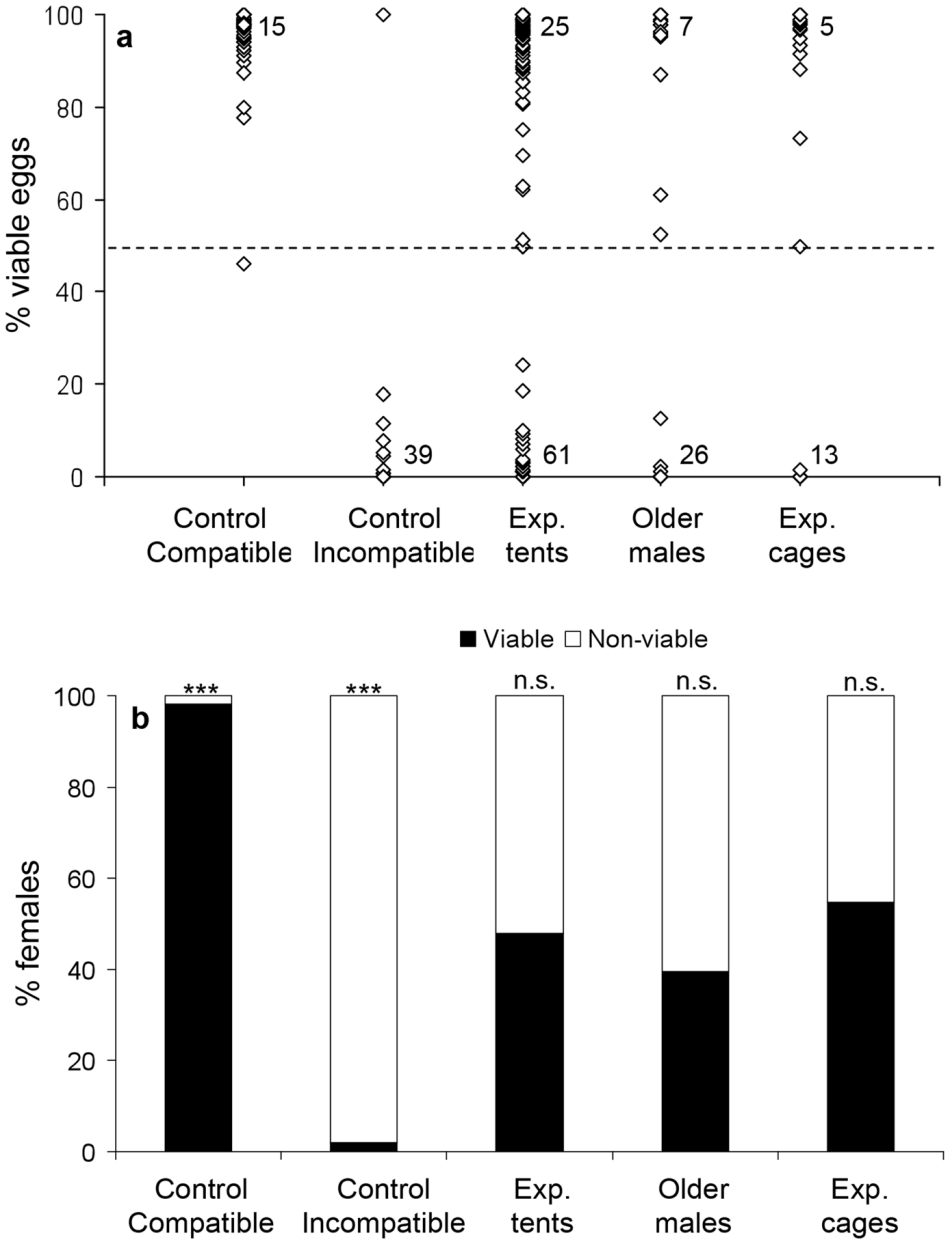
Experiment 2: Competitiveness of *w*MelPop infected vs. uninfected males. Data pooled for all females of a certain treatment. a) Percentage of viable eggs for females of compatible controls (n = 55 females), incompatible controls (n = 48 females), experimental tents (n = 158 females), experimental tents with older males (n = 48 female), and experimental semi-field cages (n = 31 females). Numbers on bottom and top of figure represent the number of overlapping data points with extreme values (0% and 100% respectively). The dashed line represent the threshold for scoring females as viable (≥50%) or non-viable (<50%). b) Percentage of viable and non-viable females in each of the above treatments. Asterisks represent significance level for deviation from 1∶1 using observed vs. expected chi square test with each tent/cage as a repetition (see text).

The chi square test which considered each trial as a repetition indicated no deviation from 50% in the percentage of viable vs. non-viable females in the experimental tents (Fig. 2, χ^2^ = 6.03, df = 11, p = 0.87, n = 12 tents, 158 females) and semi-field cages (Fig. 2, χ^2^ = 0.87, df = 2, p = 0.65, n = 3 cages, 31 females), suggesting similar competitiveness for *w*MelPop infected and uninfected males. In addition, there was no deviation from 50% in the tents with older infected and uninfected males (Fig. 2, χ^2^ = 1.18, df = 2, p = 0.55, n = 3 tents, 48 females). The competitiveness index of infected vs. uninfected males was 1.08 for males competing in the tents, 0.81 in the cages and 1.54 for older infected vs. older uninfected males. There was no significant difference in the frequency of viable vs. non-viable females when using younger or older males (Fisher's exact test based on pooled data, P = 0.33). A single female of the compatible control produced less than 50% viable eggs (Fig. 2, χ^2^ = 25.67, df = 4, p<0.001, n = 5 tents, 55 females) and a single female of the incompatible control produced 100% viable eggs (Fig. 2, χ^2^ = 22.22, df = 3, p<0.001, n = 5 tents, 48 females). This was unexpected for an incompatible cross and may represent a contaminant.

### Competitiveness of wMel infected vs. uninfected males of different sizes—Exp. 3

**Figure 3:**
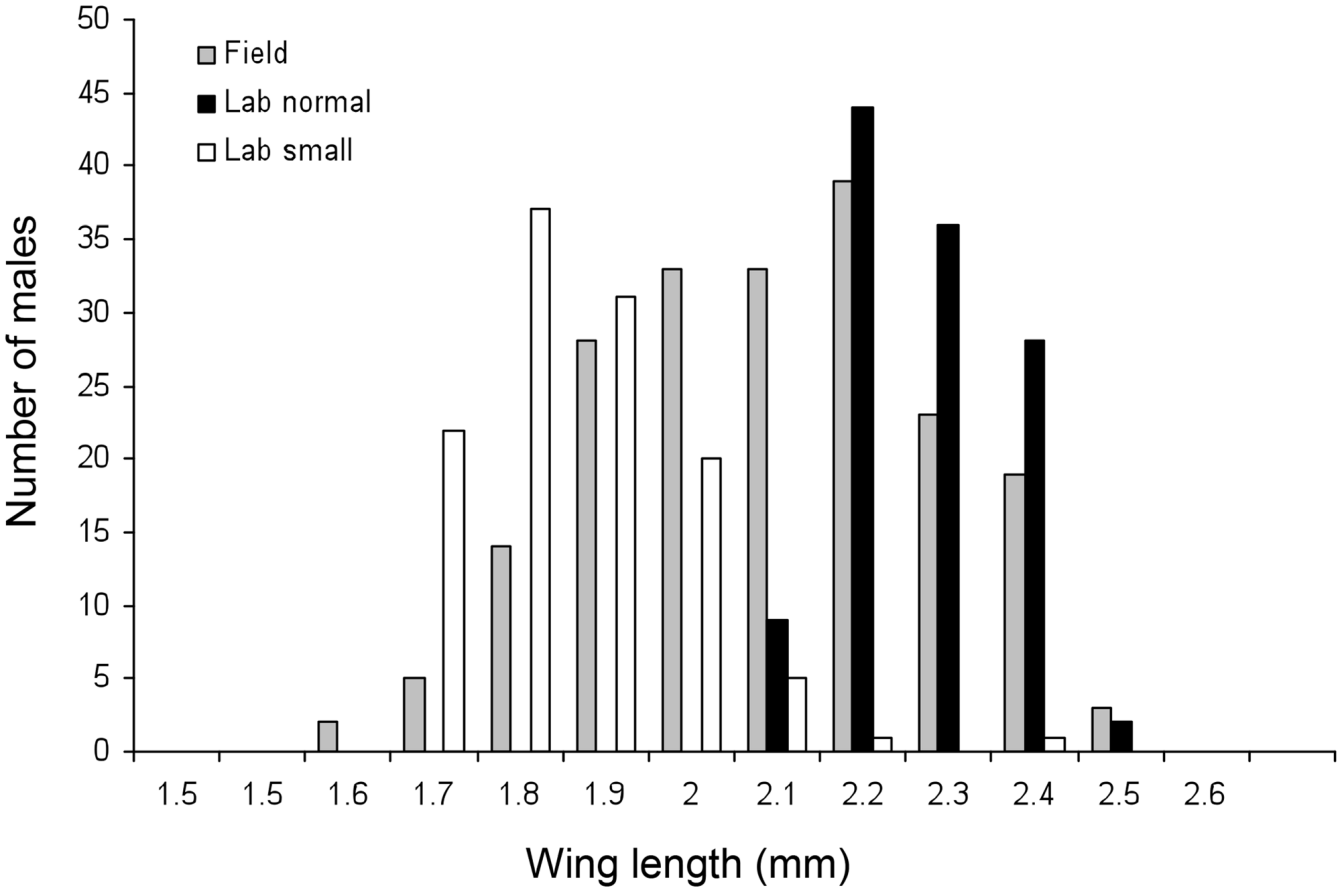
Male body size in the different rearing groups. Distribution male body size (estimated as wing length) for a sample of large lab reared males (fed *ad libitum*, n = 119), small lab reared males (fed 1/4 the amount of food, n = 117) and males trapped from the field during Nov-Dec 2012 (n =  males).

**Figure 4:**
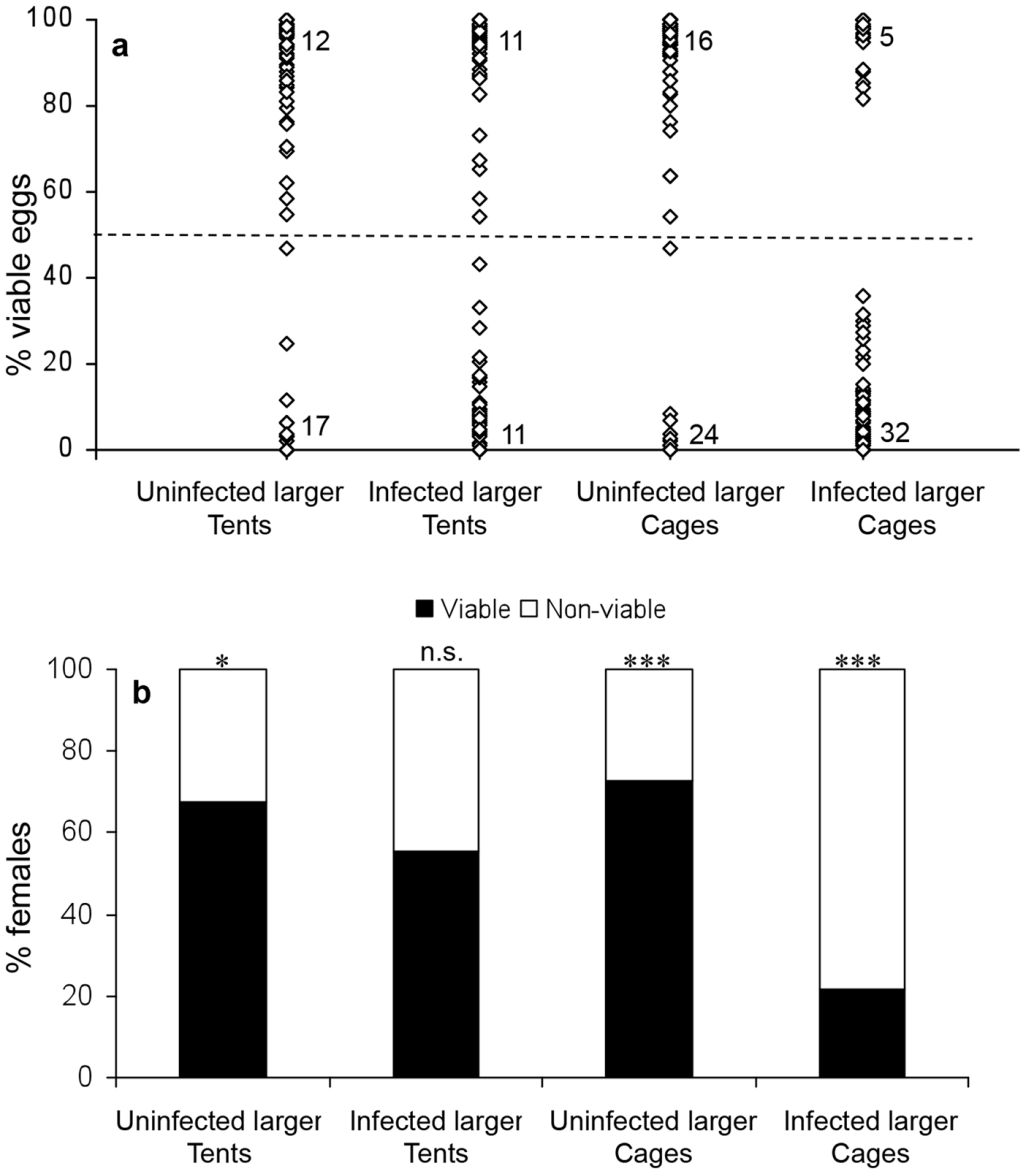
Experiment 3: Competitiveness of *w*Mel infected vs. uninfected males of different sizes. Data pooled for all females of a certain treatment. a) Percentage of viable eggs for females of tents with larger uninfected males (n = 86 females), tents with larger infected males (n = 93 females), semi-field cages with larger uninfected males (n =  females) and semi-field cages with larger infected males (n =  females). Numbers on bottom and top of figure represent the number of overlapping data points with extreme values (0% and 100% respectively). The dashed line represent the threshold for scoring females as viable (≥50%) or non-viable (<50%). b) Percentage of viable and non-viable females in each of the above treatments. Asterisks represent significance level for deviation from 1∶1 using observed vs. expected chi square test with each tent/cage as a repetition (see text).

Males reared on a low nutrition diet were within the lower range of the size distribution for wild males, while males fed *ad libitum* were within the higher range (Fig. 3). Male size differed significantly among the groups (two-way ANOVA, F_2,429_ = 81.41, p<0.001; means ± SD (n) were 1.83±0.12 mm (117) for small males, 2.14±.09 mm (119) for large males, 2.04±.19 (199) for wild males), but this was not affected by infection status (F_1,429_ = 0.91, p = 0.34) or by the interaction between rearing condition and infection status (F_2,429_ = 0.57, p = 0.57). The results from the experimental tents were not consistent: while most of the females from tents with larger uninfected males produced viable eggs, suggesting an advantage to larger males (Fig. 4, χ^2^ = 11.30, df = 5, p = 0.05, n = 6 tents, 86 females), in the tents with larger infected males the percentage of females producing viable eggs did not deviate from 50% (Fig. 4, χ^2^ = 6.31, df = 5, p = 0.28, n = 6 tents, 93 females) and the proportion of viable vs. non-viable females did not differ significantly between these treatments (Fisher exact test, p = 0.09). In contrast, the results from the semi-field cages were consistent, with higher competitiveness for larger males of either infection status: the majority of females produced viable eggs when uninfected males were larger (Fig. 4, χ^2^  = 19.72, df = 4, p<0.001, n = 5 cages, 118 females) and the majority of females produced non-viable eggs when infected males were larger (Fig. 4, χ^2^ = 20.09, df = 4, p = 0.005, n = 5 cages, 101 females). As expected, the proportion of females mating with uninfected (viable eggs) and infected (non-viable eggs) males differed significantly between these treatments (Fisher exact test, p<0.001).

### Effect of body size, infection status and female presence of on male survival—Exp. 4

There was only a single dead male in the cages one day after the start of the experiment. After a week around 70% of males had died. Male size or infection status had no effect on the number of dead males (out of 10 per cage) (3 way ANOVA, F_1,26_ = 1.10, p = 0.30 for male-size; F_1,26_ = 0.04, p = 0.85 for infection status), but female presence significantly increased male mortality (F_1,26_ = 13.43, p<0.001; mean number of dead males ± SD  = 9.07±1.44 for cages with females and 5.60±3.22 for cages without females).

## Discussion

Understanding the reproductive biology of mosquito males is critical for designing disease control programs that rely on the mass-release of modified mosquitoes [26]. Male competitiveness is of particular interest while releasing mosquitoes infected by the virus-blocking bacterium *Wolbachia*, because the ability of *Wolbachia* to spread in mosquito populations is dependent on the ability of infected males to acquire mates in the field. Despite its importance, the effect of *Wolbachia* infection on *A. aegypti* male reproductive success under natural or semi-natural conditions has not previously been examined directly. We found no evidence for fitness costs imposed by *Wolbachia* of either strain on male competitiveness, in both tents and in semi-field cages that mimic mosquito natural habitat, and we estimated relatively high competitiveness values for infected males (C = 0.7–1.54). Moreover, we found evidence for higher success of larger males in the semi-field cages, suggesting a potential advantage for artificially-reared *Wolbachia*-infected males over smaller wild males during field releases aimed at introducing *Wolbachia*.

The lack of effect of the *Wolbachia* strain *w*Mel on male competitiveness is perhaps not surprising. The *w*Mel strain does not over-replicate in mosquito cells and was shown to induce only minor fitness costs under both laboratory and semi-field conditions [19]. Low fitness costs were also inferred by *Wolbachia's* ability to establish and persist following a mass-release in several locations [20], although it is yet to be determined to what extent the infection can spread beyond the release zones. Moreover, the *w*Mel line that was used in the current experiments was backcrossed with wild strains regularly and hence no fitness costs due to reduced genetic diversity or laboratory adaptation were expected. In contrast, the *w*MelPop strain is known to induce high fitness costs to *A. aegypti* in the laboratory and in semi-field cages. Following a release, *w*MelPop-infected females had lower oviposition success compared to wild females [30], but the relative success of infected males in the field was not examined. Finally, because some fitness effects are more likely to be expressed at an older age (*e.g.*, reduced biting ability in females [38] and reduced metabolic rate in males [24]), we also ran experiments with infected vs. uninfected older males (see methods). However, none of the results comparing competitiveness of *w*Melpop infected males to uninfected males suggested a reduced ability of infected males to acquire mates. These findings are consistent with previous results showing a lack of effect of the *Wolbachia* infection on male competitiveness in *A. polynesiensis* [41], [42] or *A. albopictus* [43], [44] under field and semi-field conditions, and relatively high competitiveness values for infected males (*e.g.*, 0.68 [42];, 0.84–0.92 [41] and 0.95–1.04 [43]). In addition, *Wolbachia* had no effect on sperm quality or on the ability of male *A. aegypti* to successfully mate with multiple females under laboratory conditions [21]. Hence, in contrast to alternative control strategies that were shown to reduce male competitiveness [45], [46], [47]; the use of *Wolbachia* does not seem to compromise male performance.

Despite the consistent results and the experimental conditions that may partly reflect conditions experienced by *A. aegypti*, some *Wolbachia* effects might have been overlooked in this study. First, the density of mosquitoes in the experiments was within the higher range of densities observed for mosquitoes in the field [34], [35]. The space in the tents, and to a lesser extent in the semi-field cages, was less than normally available to wild mosquitoes. *Aedes aegypti* often mate locally and exhibit limited dispersal, but they may potentially move a few hundred meters [48]. Higher densities and limited space might simultaneously intensify some aspects of competition (*e.g.*, male-male direct interactions) while relaxing others (*e.g.*, ability to locate females). Second, we did not test the mating competitiveness of even older males, partly because *Aedes* mosquitoes may not survive particularly long in the field [34], [49]. Third, females in our experiments came from a line that had not been exposed to *Wolbachia* and therefore ignores assortative mating based on infection status. Even if *Wolbachia* does not impose direct effect on male performance, uninfected females from populations previously exposed to *Wolbachia* (*e.g.*, from around release zones) might evolve discrimination against infected males to avoid the high costs of incompatibility (*i.e.*, the production of non-viable eggs), as predicted by theory [50]. It might be worthwhile to study *Wolbachia* effects directly in the field during and at different intervals following a mass release by repeatedly comparing the proportion of infected males among those that are sexually active (captured from swarms or directly during copulation) to their estimated proportion in the population (*e.g.*, from resting population or trap collections).

One factor that may determine the competitiveness of infected vs. uninfected males at the time of releases is their relative body size. Lab-reared mosquitoes released in control programs are likely to be larger, and hence potentially more competitive than wild mosquitoes. In agreement with this expectation, we found that *A. aegypti* males fed on a high nutrition diet in the lab were larger than those from a low nutrition diet and males from the field (though, to a lesser extent). These results match findings obtained previously for females, which also showed that large females may have a reproductive advantage under field conditions [39]. Our results support the hypothesis that larger well-fed males have higher competitiveness compared to smaller underfed males. Because mating behavior of individual mosquitoes was not observed directly under the experimental conditions, the mechanism responsible for the size advantage is unknown. Larger males could have potentially been more successful in locating females, competing with other males, attracting females, copulating with females or fertilizing their eggs [26]. Assortative mating based on size could have potentially further contributed to the advantage of larger males in mating with lab-reared females. This could be further investigated by manipulating female size as well as male size. The size advantage was evident in the semi-field cages, but not clearly in the experimental tents, which may indicate that large size increases male mobility and ability to locate females in a large arena.

In many mosquito species, seminal fluids transmitted by males to females during mating result in reduced female sexual receptivity and hence female monogamy [36], [37]. Nevertheless, evidence exists for some degree of multiple mating in *A. aegypti* [26], [51]. Sperm competition and cryptic female choice could therefore play an additional role in determining male reproductive success [52]. The majority of females in the current experiments produced more than 90% or less than 10% viable eggs, similar to the controls, suggesting that they had mated with a single male (or possibly with multiple males of a similar infection status). However, some females produced intermediate egg viability levels of 10%–90%, suggesting the possibility of occasional multiple inseminations (see Figs. 1a, 2a and 4a). The percentage of females producing intermediate egg viability levels (14% in experiment 1, 11% in experiment 2 and 20% in experiment 3) was similar to a previous report on the incidence of multiple mating in *A. aegypti* (14% of females [53]). The higher occurrence in experiment 3 (male size experiment) might reflect a higher tendency of females to remate after mating to a smaller male, or by a seasonal increase in temperatures and hence higher activity levels of the mosquitoes during this experiment (conducted in the hotter months- Nov 2013-Feb 2014). Further tests are required to determine the exact level of multiple mating under experimental and field conditions, and the relative importance of sperm competition and cryptic female choice in determining male mating success.

In conclusion, *Wolbachia* does not seem to compromise male competitiveness, and infected males might even have an advantage over wild males during a field release episode. The lack of *Wolbachia* effect under the experimental conditions opens up an opportunity to study additional factors that might influence male success (similar to the body-size experiment in the current study). For example, infected and uninfected males can be used to study the competitiveness of males of different ages, mating histories, rearing conditions etc. Further work should aim at studying *Wolbachia* effects directly in the field during and at different intervals following a release, while taking male size into account. In addition, mechanisms contributing to male size advantage should be further explored, for example via direct observations on the behavior of females exposed to both small and large males, and via the use of molecular techniques to determine paternity.
